# 4-[(1*RS*,5*RS*,7*SR*)-5-Methyl-2,4-dioxo-3,6-diaza­bicyclo­[3.2.1]octan-7-yl]benzonitrile

**DOI:** 10.1107/S1600536812020156

**Published:** 2012-05-16

**Authors:** Konstantin V. Kudryavtsev, Andrei V. Churakov

**Affiliations:** a Department of Chemistry, M.V. Lomonosov Moscow State University, Leninskie Gory 1/3, Moscow 119991, Russian Federation; b Institute of Physiologically Active Compounds, Russian Academy of Sciences, Chernogolovka 142432, Moscow region, Russian Federation; c Institute of General and Inorganic Chemistry, Russian Academy of Sciences, Leninskii prosp. 31, Moscow 119991, Russian Federation

## Abstract

In the title compound, C_14_H_13_N_3_O_2_, the relative stereochemistry of the three stereogenic C atoms has been determined. In the crystal, N—H⋯O hydrogen bonds link the mol­ecules into chains of inversion dimers running along the *b* axis.

## Related literature

For general background to chemistry affording a bridged 3,6-diaza­bicyclo­[3.2.1]octane scaffold, substituted at the 3, 5, 6, and 7 positions, and the biological activity of this class of compounds, see: Kudryavtsev (2010 ▶).
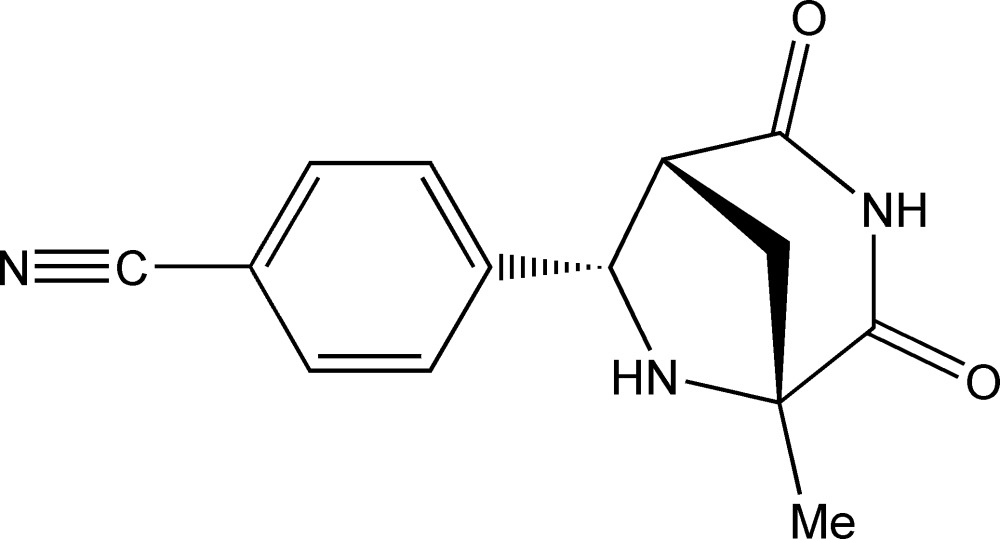


## Experimental

### 

#### Crystal data

C_14_H_13_N_3_O_2_
*M* *_r_* = 255.27
Monoclinic, 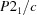

*a* = 14.8572 (13) Å
*b* = 6.2269 (6) Å
*c* = 13.1215 (12) Å
β = 95.568 (1)°
*V* = 1208.20 (19) Å^3^
*Z* = 4
Mo *K*α radiation
μ = 0.10 mm^−1^
*T* = 150 K
0.40 × 0.15 × 0.10 mm

#### Data collection

Bruker SMART APEXII diffractometer
Absorption correction: multi-scan (*SADABS*; Bruker, 2008 ▶) *T*_min_ = 0.962, *T*_max_ = 0.990
11909 measured reflections
2924 independent reflections
2601 reflections with *I* > 2σ(*I*)
*R*_int_ = 0.019

#### Refinement

*R*[*F* ^2^ > 2σ(*F* ^2^)] = 0.040
*wR*(*F* ^2^) = 0.110
*S* = 1.06
2924 reflections
224 parameters
All H-atom parameters refined
Δρ_max_ = 0.35 e Å^−3^
Δρ_min_ = −0.21 e Å^−3^

### 

Data collection: *APEX2* (Bruker, 2008 ▶); cell refinement: *SAINT* (Bruker, 2008 ▶); data reduction: *SAINT*; program(s) used to solve structure: *SHELXTL* (Sheldrick, 2008 ▶); program(s) used to refine structure: *SHELXTL*; molecular graphics: *SHELXTL*; software used to prepare material for publication: *SHELXTL*.

## Supplementary Material

Crystal structure: contains datablock(s) I, global. DOI: 10.1107/S1600536812020156/ff2066sup1.cif

Structure factors: contains datablock(s) I. DOI: 10.1107/S1600536812020156/ff2066Isup2.hkl

Supplementary material file. DOI: 10.1107/S1600536812020156/ff2066Isup3.cml

Additional supplementary materials:  crystallographic information; 3D view; checkCIF report

## Figures and Tables

**Table 1:** Hydrogen-bond geometry (Å, °)

*D*—H⋯*A*	*D*—H	H⋯*A*	*D*⋯*A*	*D*—H⋯*A*
N2—H2⋯O2^i^	0.899 (18)	2.368 (19)	3.2377 (14)	162.8 (15)
N1—H1⋯O1^ii^	0.877 (17)	2.032 (17)	2.9019 (14)	171.3 (15)

Symmetry codes: (i) 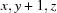; (ii) 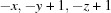.

**Fig. 1.**
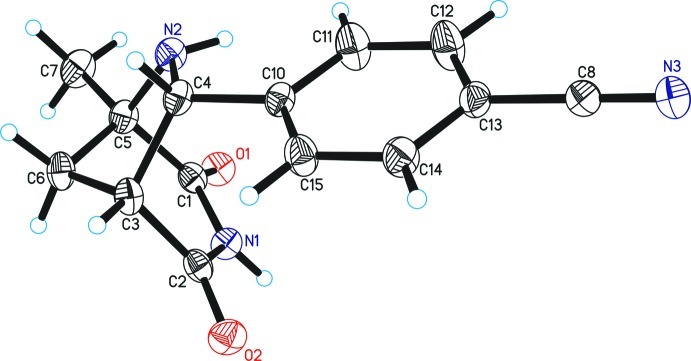
The molecular structure of the title compound, showing the numbering scheme adopted. Displacement ellipsoids are shown at the 50% probability level.

**Fig. 2.**
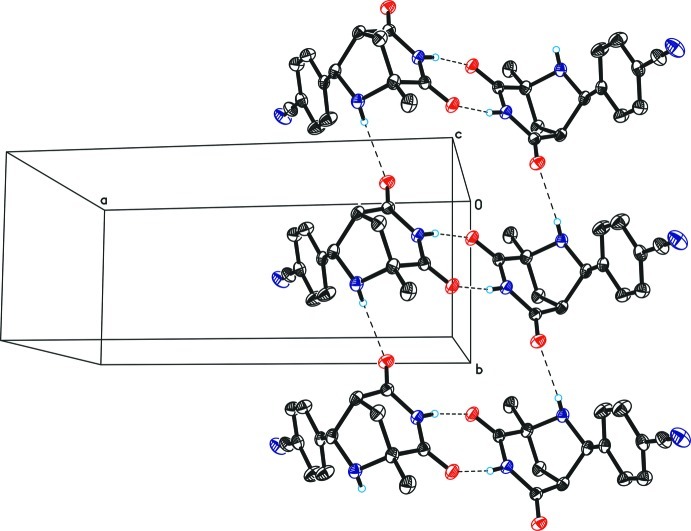
Hydrogen-bonded chains along b-axis in the structure of the title compound. H-bonds are shown as dashed lines.

**Fig. 3.**
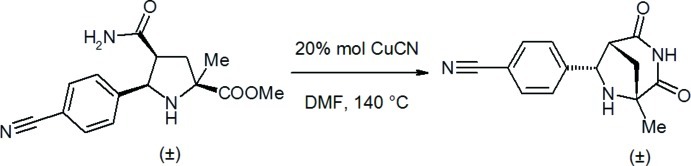
Synthetic scheme.

## supplementary crystallographic information

### Comment

In the title compound, 4-cyanophenyl substituent occupies *endo* position (Fig. 1). The –C(=O)NHC(=O)- system is planar within 0.046 (3) Å. The adjacent molecules are combined into double centrosymmetric chains along *b*-axis by N—H···O=C hydrogen bonds (Fig. 2). These chains are linked by weak van der Waals interactions.

3,6-Diazabicyclo[3.2.1]octanes are of interest as a structural motif for enzymes inhibitors. Synthesis of substituted 3,6-diazabicyclo[3.2.1]octane is based on copper(I) catalyzed intramolecular imide formation (Kudryavtsev (2010), Fig. 3).

### Experimental

(2*SR*,4*SR*,5*RS*)-Methyl 4-carbamoyl-5-(4-cyanophenyl)-2-methylpyrrolidine-2-carboxylate (0.862 g, 3.0 mmol) was dissolved in 30 ml of DMF, 0.054 g (0.6 mmol) of CuCN were added, and the mixture was stirred under argon at 413 K during 6 h. The solvent was distilled off under reduced pressure, and the residue was dissolved in 20 ml of AcOEt, washed with saturated solution of NaHCO_3_ (2 *x* 7 ml). Organic phase was dried under Na_2_SO_4_, concentrated and recrystallized from hexane–ethyl acetate. 4-((1*RS*,5*RS*,7*SR*- 5-methyl-2,4-dioxo-3,6-diazabicyclo[3.2.1]octan-7-yl)benzonitrile. Yield 0.490 g (64%), colourless crystals, m.p. 497–499 K. ^1^H NMR (400 MHz, DMSO-d_6_): *δ* 1.38 (s, 3H, CH_3_), 2.00 (dd, *J* 11.8, 4.0, 1H, H-8a), 2.31 (d, *J* 11.8, 1H, H-8 b), 3.37 (br.s, 1H, H-1), 3.59 (d, *J* 7.8, 1H, N(6)H), 4.93 (dd, *J* 7.8, 5.8, 1H, H-7), 7.55 (d, *J* 8.3, 2H, Ar), 7.73 (d, *J* 8.3, 2H, Ar), 10.42(s, 1H, N(3)H). ^13^C NMR (100 MHz, DMSO-d_6_): *δ* 18.73, 40.82, 52.78, 62.73, 63.09, 109.99, 119.42, 128.01 (2 C), 132.33 (2 C), 147.52, 174.04, 176.39. Anal. Calcd. for C_14_H_13_N_3_O_2_: C, 65.87; H, 5.13; N, 16.46. Found: C, 65.92; H, 5.17; N,16.63. The crystals were obtained by slow evaporation of saturated solution in hexane–ethyl acetate (2:3) mixture at ambient temperature.

### Refinement

All hydrogen atoms were located in a difference Fourier map and refined with isotropic thermal parameters.

### Crystal data

**Table:**

C_14_H_13_N_3_O_2_	*F*(000) = 536
*M**_r_* = 255.27	*D*_x_ = 1.403 Mg m^−^^3^
Monoclinic, *P*2_1_/*c*	Mo *K*α radiation, λ = 0.71073 Å
Hall symbol: -P 2ybc	Cell parameters from 5466 reflections
*a* = 14.8572 (13) Å	θ = 2.3–31.0°
*b* = 6.2269 (6) Å	µ = 0.10 mm^−^^1^
*c* = 13.1215 (12) Å	*T* = 150 K
β = 95.568 (1)°	Block, colourless
*V* = 1208.20 (19) Å^3^	0.40 × 0.15 × 0.10 mm
*Z* = 4	

### Data collection

**Table:**

Bruker SMART APEXII diffractometer	2924 independent reflections
Radiation source: fine-focus sealed tube	2601 reflections with *I* > 2σ(*I*)
Graphite monochromator	*R*_int_ = 0.019
ω scans	θ_max_ = 28.0°, θ_min_ = 2.8°
Absorption correction: multi-scan (*SADABS*; Bruker, 2008)	*h* = −19→19
*T*_min_ = 0.962, *T*_max_ = 0.990	*k* = −8→8
11909 measured reflections	*l* = −17→17

### Refinement

**Table:**

Refinement on *F*^2^	Primary atom site location: structure-invariant direct methods
Least-squares matrix: full	Secondary atom site location: difference Fourier map
*R*[*F*^2^ > 2σ(*F*^2^)] = 0.040	Hydrogen site location: difference Fourier map
*wR*(*F*^2^) = 0.110	All H-atom parameters refined
*S* = 1.06	*w* = 1/[σ^2^(*F*_o_^2^) + (0.0568*P*)^2^ + 0.4568*P*] where *P* = (*F*_o_^2^ + 2*F*_c_^2^)/3
2924 reflections	(Δ/σ)_max_ < 0.001
224 parameters	Δρ_max_ = 0.35 e Å^−^^3^
0 restraints	Δρ_min_ = −0.21 e Å^−^^3^

### Special details

**Table:**

Geometry. All e.s.d.'s (except the e.s.d. in the dihedral angle between two l.s. planes) are estimated using the full covariance matrix. The cell e.s.d.'s are taken into account individually in the estimation of e.s.d.'s in distances, angles and torsion angles; correlations between e.s.d.'s in cell parameters are only used when they are defined by crystal symmetry. An approximate (isotropic) treatment of cell e.s.d.'s is used for estimating e.s.d.'s involving l.s. planes.
Refinement. Refinement of *F*^2^ against ALL reflections. The weighted *R*-factor *wR* and goodness of fit *S* are based on *F*^2^, conventional *R*-factors *R* are based on *F*, with *F* set to zero for negative *F*^2^. The threshold expression of *F*^2^ > σ(*F*^2^) is used only for calculating *R*-factors(gt) *etc*. and is not relevant to the choice of reflections for refinement. *R*-factors based on *F*^2^ are statistically about twice as large as those based on *F*, and *R*- factors based on ALL data will be even larger.

### Fractional atomic coordinates and isotropic or equivalent isotropic displacement parameters (Å^2^)

**Table:**

	*x*	*y*	*z*	*U*_iso_*/*U*_eq_	
O1	0.01850 (6)	0.66158 (14)	0.61099 (7)	0.0262 (2)	
O2	0.17560 (6)	0.06107 (15)	0.54975 (7)	0.0271 (2)	
N1	0.10075 (7)	0.36899 (17)	0.57715 (8)	0.0213 (2)	
N2	0.23232 (7)	0.66162 (17)	0.70043 (8)	0.0218 (2)	
N3	0.47535 (9)	0.5189 (2)	0.21483 (9)	0.0350 (3)	
C1	0.08167 (7)	0.54263 (19)	0.63735 (9)	0.0205 (2)	
C2	0.16886 (8)	0.22065 (19)	0.60194 (9)	0.0203 (2)	
C3	0.23153 (8)	0.27578 (19)	0.69563 (9)	0.0208 (2)	
C4	0.28953 (8)	0.47800 (19)	0.67574 (8)	0.0202 (2)	
C5	0.14651 (8)	0.5781 (2)	0.73407 (9)	0.0218 (2)	
C6	0.17531 (8)	0.3600 (2)	0.77908 (9)	0.0237 (3)	
C7	0.10602 (9)	0.7300 (2)	0.80748 (11)	0.0302 (3)	
C8	0.44462 (9)	0.5099 (2)	0.29149 (9)	0.0262 (3)	
C10	0.32690 (7)	0.48798 (19)	0.57236 (8)	0.0193 (2)	
C11	0.32273 (10)	0.6744 (2)	0.51302 (11)	0.0326 (3)	
C12	0.36129 (10)	0.6806 (2)	0.42077 (11)	0.0340 (3)	
C13	0.40462 (8)	0.5001 (2)	0.38726 (9)	0.0227 (3)	
C14	0.41036 (9)	0.3136 (2)	0.44601 (10)	0.0257 (3)	
C15	0.37150 (9)	0.3091 (2)	0.53770 (10)	0.0251 (3)	
H4	0.3428 (10)	0.470 (2)	0.7280 (12)	0.023 (4)*	
H62	0.2141 (10)	0.378 (3)	0.8439 (12)	0.027 (4)*	
H3	0.2689 (11)	0.147 (3)	0.7158 (12)	0.029 (4)*	
H1	0.0636 (11)	0.346 (3)	0.5222 (13)	0.027 (4)*	
H61	0.1244 (10)	0.265 (2)	0.7877 (11)	0.021 (3)*	
H14	0.4399 (11)	0.192 (3)	0.4254 (13)	0.031 (4)*	
H73	0.0524 (12)	0.658 (3)	0.8305 (14)	0.039 (5)*	
H15	0.3758 (12)	0.181 (3)	0.5765 (13)	0.038 (4)*	
H2	0.2219 (11)	0.755 (3)	0.6484 (14)	0.035 (4)*	
H72	0.0885 (11)	0.867 (3)	0.7734 (13)	0.035 (4)*	
H71	0.1509 (12)	0.751 (3)	0.8687 (15)	0.042 (5)*	
H12	0.3569 (14)	0.814 (3)	0.3768 (16)	0.055 (6)*	
H11	0.2931 (13)	0.803 (3)	0.5349 (14)	0.043 (5)*	

### Atomic displacement parameters (Å^2^)

**Table:**

	*U*^11^	*U*^22^	*U*^33^	*U*^12^	*U*^13^	*U*^23^
O1	0.0212 (4)	0.0265 (5)	0.0309 (5)	0.0070 (3)	0.0025 (3)	−0.0009 (4)
O2	0.0292 (5)	0.0226 (4)	0.0297 (5)	0.0053 (4)	0.0045 (4)	−0.0035 (4)
N1	0.0191 (5)	0.0226 (5)	0.0221 (5)	0.0035 (4)	0.0016 (4)	−0.0014 (4)
N2	0.0200 (5)	0.0243 (5)	0.0219 (5)	0.0024 (4)	0.0064 (4)	−0.0021 (4)
N3	0.0419 (7)	0.0385 (7)	0.0266 (6)	−0.0124 (5)	0.0137 (5)	−0.0029 (5)
C1	0.0175 (5)	0.0220 (5)	0.0231 (5)	0.0011 (4)	0.0072 (4)	0.0012 (4)
C2	0.0198 (5)	0.0200 (5)	0.0219 (5)	0.0018 (4)	0.0070 (4)	0.0037 (4)
C3	0.0198 (5)	0.0238 (6)	0.0193 (5)	0.0054 (4)	0.0049 (4)	0.0031 (4)
C4	0.0175 (5)	0.0259 (6)	0.0174 (5)	0.0035 (4)	0.0029 (4)	−0.0009 (4)
C5	0.0200 (5)	0.0262 (6)	0.0200 (5)	0.0033 (4)	0.0062 (4)	−0.0016 (4)
C6	0.0240 (6)	0.0293 (6)	0.0189 (5)	0.0042 (5)	0.0079 (4)	0.0026 (5)
C7	0.0281 (6)	0.0365 (7)	0.0275 (6)	0.0063 (6)	0.0101 (5)	−0.0071 (6)
C8	0.0272 (6)	0.0288 (6)	0.0234 (6)	−0.0068 (5)	0.0055 (5)	−0.0021 (5)
C10	0.0160 (5)	0.0249 (6)	0.0172 (5)	0.0012 (4)	0.0027 (4)	−0.0008 (4)
C11	0.0405 (7)	0.0283 (7)	0.0311 (7)	0.0127 (6)	0.0146 (6)	0.0055 (5)
C12	0.0424 (8)	0.0315 (7)	0.0301 (7)	0.0107 (6)	0.0130 (6)	0.0108 (6)
C13	0.0204 (5)	0.0297 (6)	0.0184 (5)	−0.0040 (5)	0.0041 (4)	−0.0008 (5)
C14	0.0283 (6)	0.0246 (6)	0.0256 (6)	0.0024 (5)	0.0103 (5)	−0.0021 (5)
C15	0.0296 (6)	0.0234 (6)	0.0238 (6)	0.0053 (5)	0.0093 (5)	0.0032 (5)

### Geometric parameters (Å, º)

**Table:**

O1—C1	1.2190 (14)	C5—C6	1.5257 (17)
O2—C2	1.2164 (15)	C6—H62	0.986 (16)
N1—C1	1.3845 (15)	C6—H61	0.976 (15)
N1—C2	1.3853 (15)	C7—H73	0.986 (19)
N1—H1	0.877 (17)	C7—H72	0.988 (18)
N2—C4	1.4793 (15)	C7—H71	1.002 (19)
N2—C5	1.4831 (15)	C8—C13	1.4426 (16)
N2—H2	0.899 (18)	C10—C15	1.3944 (16)
N3—C8	1.1457 (17)	C10—C11	1.3960 (17)
C1—C5	1.5331 (16)	C11—C12	1.3890 (18)
C2—C3	1.5080 (16)	C11—H11	0.971 (19)
C3—C6	1.5327 (15)	C12—C13	1.3879 (19)
C3—C4	1.5620 (17)	C12—H12	1.01 (2)
C3—H3	0.998 (16)	C13—C14	1.3919 (18)
C4—C10	1.5161 (15)	C14—C15	1.3843 (17)
C4—H4	0.997 (15)	C14—H14	0.931 (17)
C5—C7	1.5155 (16)	C15—H15	0.944 (18)
			
C1—N1—C2	124.90 (10)	C5—C6—H62	110.7 (9)
C1—N1—H1	116.8 (11)	C3—C6—H62	109.9 (9)
C2—N1—H1	118.0 (11)	C5—C6—H61	113.2 (9)
C4—N2—C5	108.84 (9)	C3—C6—H61	110.9 (9)
C4—N2—H2	113.2 (11)	H62—C6—H61	111.2 (12)
C5—N2—H2	111.3 (11)	C5—C7—H73	107.1 (11)
O1—C1—N1	120.48 (11)	C5—C7—H72	110.8 (10)
O1—C1—C5	123.50 (11)	H73—C7—H72	110.3 (14)
N1—C1—C5	115.97 (10)	C5—C7—H71	108.5 (11)
O2—C2—N1	120.71 (11)	H73—C7—H71	107.9 (15)
O2—C2—C3	124.53 (11)	H72—C7—H71	112.1 (15)
N1—C2—C3	114.76 (10)	N3—C8—C13	179.09 (15)
C2—C3—C6	108.91 (9)	C15—C10—C11	118.62 (11)
C2—C3—C4	110.75 (9)	C15—C10—C4	119.08 (10)
C6—C3—C4	101.02 (9)	C11—C10—C4	122.20 (11)
C2—C3—H3	108.4 (9)	C12—C11—C10	120.64 (12)
C6—C3—H3	114.4 (9)	C12—C11—H11	118.1 (11)
C4—C3—H3	113.1 (9)	C10—C11—H11	121.3 (11)
N2—C4—C10	115.62 (10)	C13—C12—C11	119.78 (12)
N2—C4—C3	104.39 (9)	C13—C12—H12	119.5 (12)
C10—C4—C3	116.00 (9)	C11—C12—H12	120.7 (12)
N2—C4—H4	108.7 (9)	C12—C13—C14	120.37 (11)
C10—C4—H4	106.4 (9)	C12—C13—C8	119.00 (12)
C3—C4—H4	105.1 (9)	C14—C13—C8	120.62 (11)
N2—C5—C7	112.09 (11)	C15—C14—C13	119.33 (11)
N2—C5—C6	102.15 (9)	C15—C14—H14	119.0 (10)
C7—C5—C6	115.05 (10)	C13—C14—H14	121.7 (10)
N2—C5—C1	107.06 (9)	C14—C15—C10	121.25 (12)
C7—C5—C1	111.09 (10)	C14—C15—H15	118.2 (11)
C6—C5—C1	108.79 (10)	C10—C15—H15	120.5 (11)
C5—C6—C3	100.36 (9)		

### Hydrogen-bond geometry (Å, º)

**Table:**

*D*—H···*A*	*D*—H	H···*A*	*D*···*A*	*D*—H···*A*
N2—H2···O2^i^	0.899 (18)	2.368 (19)	3.2377 (14)	162.8 (15)
N1—H1···O1^ii^	0.877 (17)	2.032 (17)	2.9019 (14)	171.3 (15)

Symmetry codes: (i) *x*, *y*+1, *z*; (ii) −*x*, −*y*+1, −*z*+1.
